# Supplementary material for: The influence of COVID-19 risk perception and vaccination status on the number of social contacts across Europe: insights from the CoMix study
Source: BMC Public Health. 2023 Jul 13;23:1350. doi: 10.1186/s12889-023-16252-z (PMC10347859; doi:10.1186/s12889-023-16252-z)
Supplement: Supplementary file 1 — Additional file 1. [file 12889_2023_16252_MOESM1_ESM.pdf]

Additional file 1 for: "The influence of COVID-19 risk perception and vaccination status on the number of social contacts across Europe: insights from the CoMix study"

James Wambua[1*], Neilshan Loedy[1], Christopher I Jarvis[2], Kerry LM Wong[2], Christel Faes[1], Rok Grah[3], Bastian Prasse[3], Frank Sandmann[3], Rene Niehus[3], Helen Johnson[3**], W.John Edmunds[2], Philippe Beutels[4,5], Niel Hens[1,4], Pietro Coletti[1]

May 29, 2023

[1]Data Science Institute, I-BioStat, Hasselt University, Hasselt, Belgium.

[2]Centre for Mathematical Modelling of Infectious Diseases, Department of Infectious Disease Epidemiology, London School of Hygiene and Tropical Medicine, Keppel Street, WC1E 7HT London, UK.

[3]European Centre for Disease Prevention and Control (ECDC), Gustav III:s Boulevard 40, 169 73 Solna, Sweden.

[4]Centre for Health Economics Research and Modelling Infectious Diseases, Vaccine & Infectious Disease Institute, University of Antwerp, Antwerp, Belgium.

[5]The University of New South Wales, School of Public Health and Community Medicine, Sydney, NSW 2033, Australia.

** Current address: Health Emergency Preparedness and Response Authority (HERA), European Commission, 1049 Brussels, Belgium.

* Corresponding author: james.wambua@uhasselt.be

Supporting information

Country	Start date of data collection	End date of data collection	Total survey rounds in Adults	Total survey rounds in Children
Austria	22 December 2020	19 April 2021	7	2
Denmark	22 December 2020	16 April 2021	7	2
France	21 December 2020	08 April 2021	7	2
Italy	21 December 2020	23 April 2021	7	2
Portugal	22 December 2020	22 April 2021	7	2
Poland	22 December 2020	19 April 2021	7	2
Spain	21 December 2020	12 May 2021	7	2
Greece	18 February 2021	08 June 2021	7	2
Slovenia	04 March 2021	09 June 2021	7	2
Croatia	20 April 2021	18 August 2021	7	2
Estonia	20 April 2021	23 August 2021	7	2
Hungary	20 April 2021	18 August 2021	6	2
Slovakia	20 April 2021	16 August 2021	7	2
Switzerland	3 June 2021	15 September 2021	6	2
Lithuania	2 July 2021	30 September 2021	6	2
Finland	2 July 2021	30 September 2021	6	2

Supplementary Table 1: Country specific timelines of the CoMix data collection in the 16 countries included in the study.

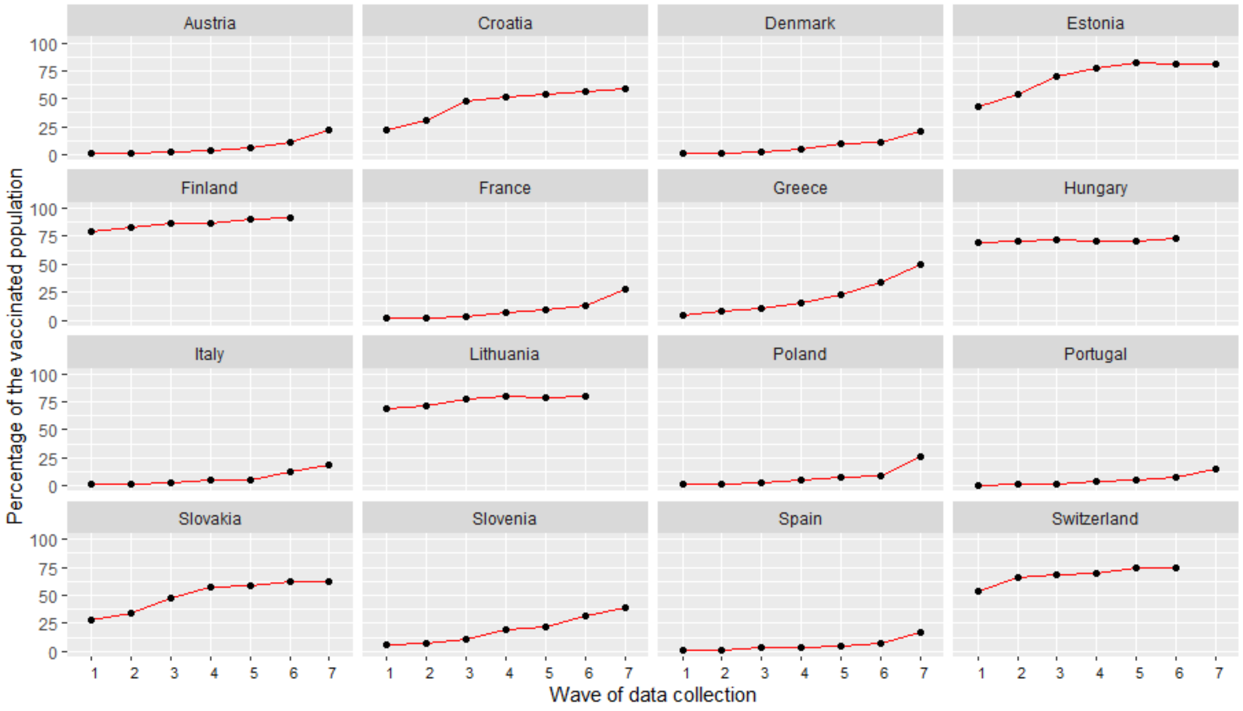

Supplementary Figure 1: Percentage of the adult population (18 years or above) vaccinated with the first dose of any of the COVID-19 vaccine during the data collection period in the different countries.

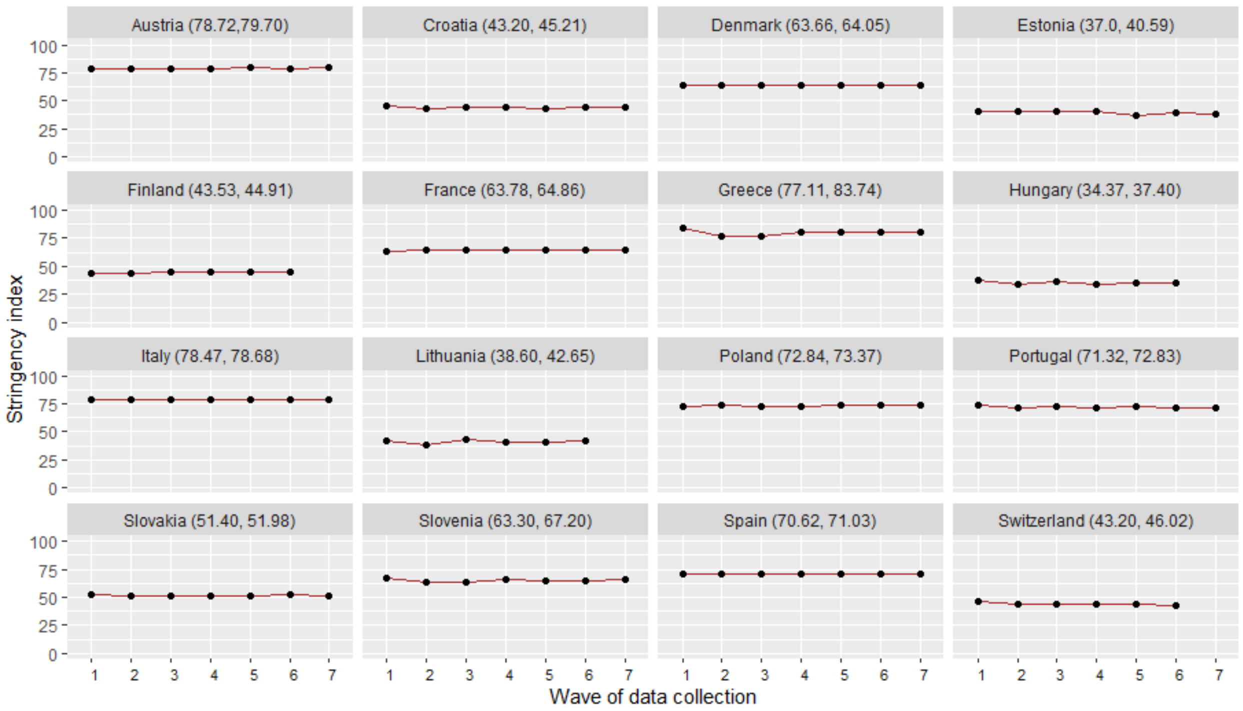

Supplementary Figure 2: Variations in the stringency index during the data collection period in the different countries.

Country	Cronbach's alpha	95% Confidence intervals
Austria	0.61	0.59 - 0.62
Croatia	0.67	0.65 - 0.68
Denmark	0.33	0.30 - 0.36
Estonia	0.67	0.66 - 0.69
Finland	0.65	0.63 - 0.66
France	0.70	0.68 - 0.71
Greece	0.74	0.73 - 0.75
Hungary	0.67	0.66 - 0.69
Italy	0.64	0.63 - 0.66
Lithuania	0.76	0.75 - 0.77
Poland	0.75	0.74 - 0.76
Portugal	0.41	0.38 - 0.43
Slovakia	0.70	0.69 - 0.71
Slovenia	0.65	0.63 - 0.66
Spain	0.56	0.54 - 0.58
Switzerland	0.66	0.65 - 0.68

Supplementary Table 2: Cronbach's alpha for each individual country.

0.1 Reliability analysis for internal consistency

The risk perceptions consisted of three statements that the participants were asked to respond to. The first statement was *"I am likely to catch coronavirus"*, the second was *"I am worried that I*

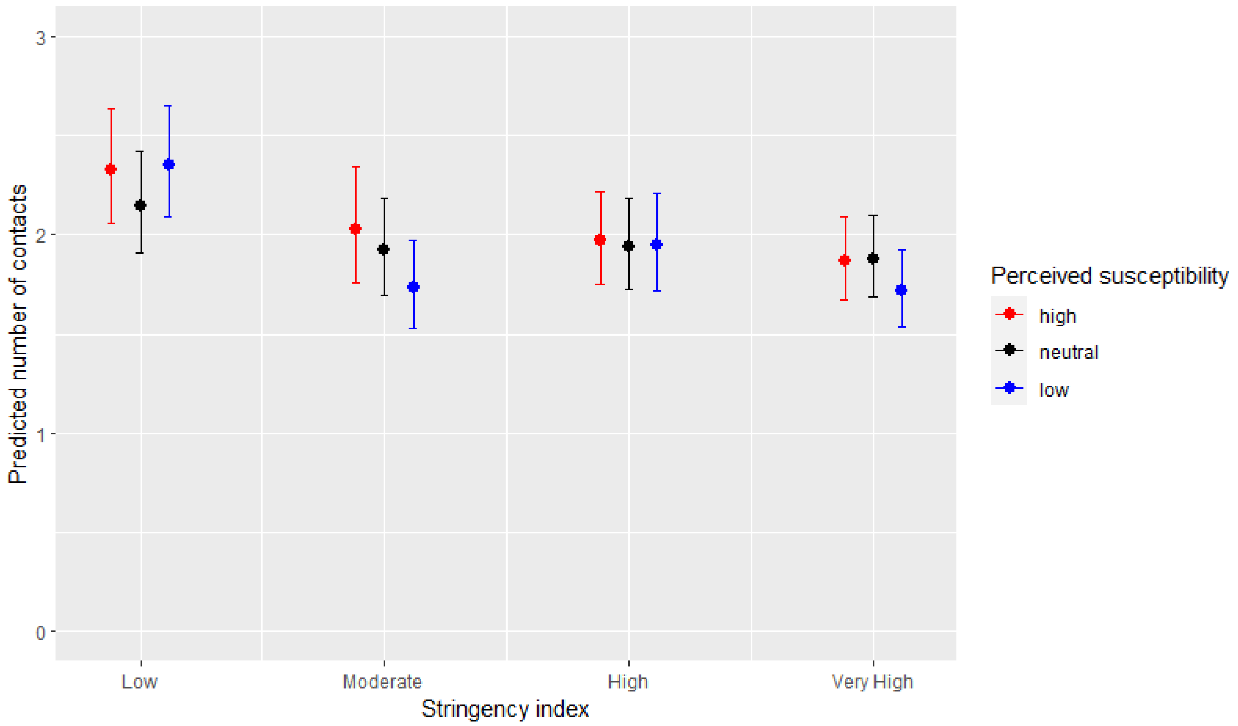

Supplementary Figure 3: Predicted number of contacts by perceived susceptibility and stringency index with 95% CI for the perceived susceptibility model in the period between December 2020 and September 2021.

might spread coronavirus to someone who is vulnerable", and the third was *"coronavirus would be a serious illness for me"*. These items were coded on 5-point Likert scales. The response option *"do not know"* was treated as a missing value in the reliability analysis, but was included in the modeling analysis as explained below.

We utilized Cronbach's alpha as the reliability measure of internal consistency of the Likert-scales of the risk perception items [1]. We considered a threshold of 0.80 of the Cronbach's alpha reliability coefficient to group the items [1]. The three risk perceptions items had an overall Cronbach's alpha of 0.670 (95% confidence interval (CI) 0.669 - 0.671) for all the countries combined. The Cronbach's alpha for each individual country ranged between 0.33 (95% CI 0.30 - 0.36) and 0.76 (95% CI 0.75 - 0.77). More information on the Cronbach's alpha for each country is detailed in Supplementary Table 2. Thus, following the resulting overall Cronbach's alpha for the three items which was below the considered threshold, we did not combine the items into one composite variable to represent the underlying risk perception construct. Therefore, the three risk perception items were considered to measure three separate constructs. We refer to these constructs as, *perceived severity* for the item *"coronavirus would be a serious illness for me"*, *perceived susceptibility* for the item *"I am likely to catch coronavirus"*, and *perceived risk to the vulnerable* for the item *"I am worried that I might spread coronavirus to someone who is vulnerable"*. Motivated by exploratory analyses, we categorised the 6 response options into three response levels: low perception ('tend to disagree' and 'strongly disagree'), neutral ('neither agree nor disagree' and 'don't know') and high perception ('strongly agree' and 'tend to agree').

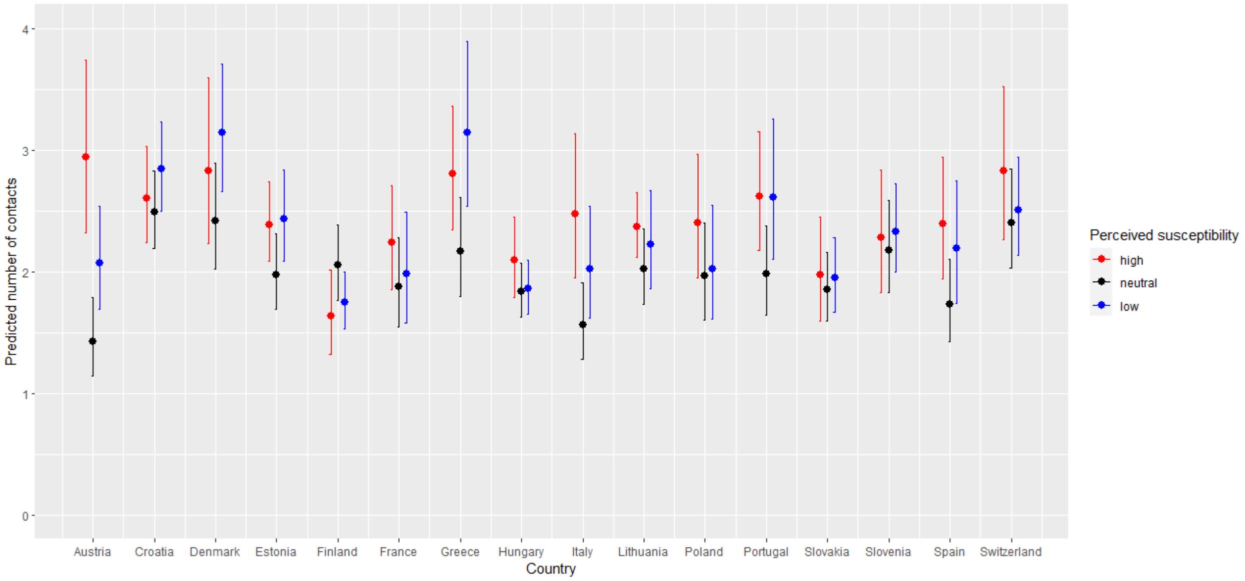

Supplementary Figure 4: Predicted number of contacts from the marginal effects of interaction between perceived susceptibility and country with 95% CI for the perceived susceptibility model with country as a fixed effect in the period between December 2020 and September 2021.

References

[1] Gliem, J.A., Gliem, R.R.: Calculating, interpreting, and reporting cronbach's alpha reliability coefficient for Likert-type scales. (2003). Midwest Research-to-Practice Conference in Adult, Continuing, and Community Education

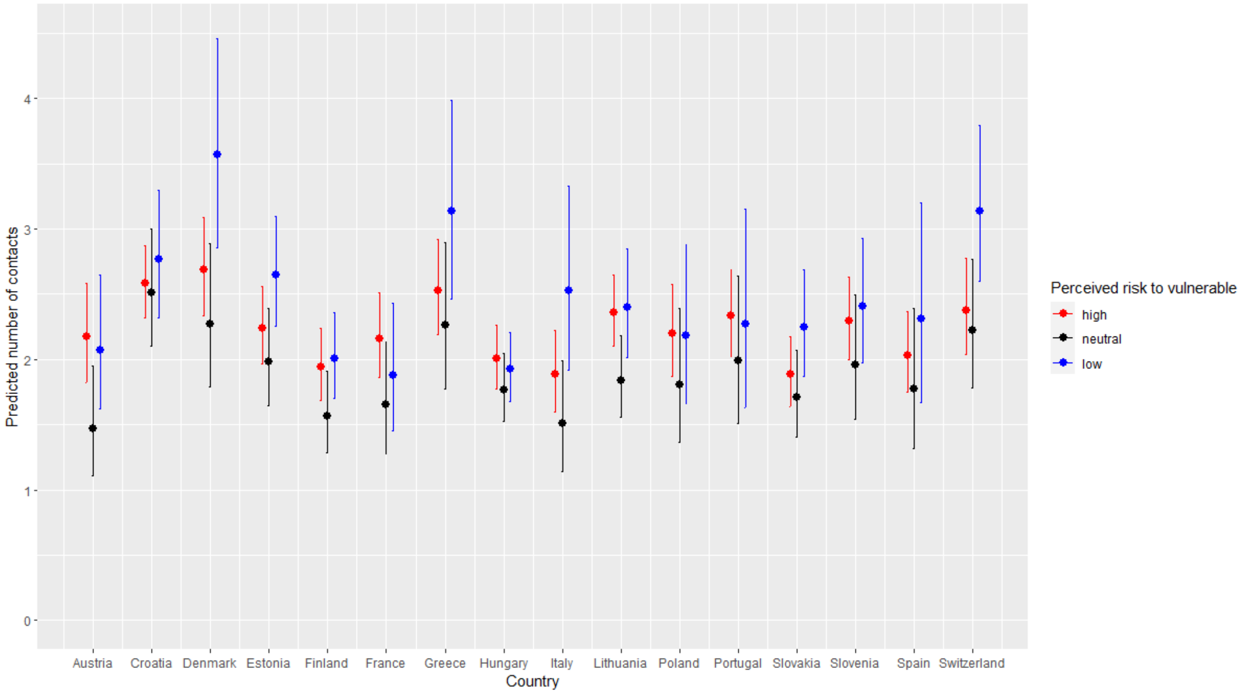

Supplementary Figure 5: Predicted number of contacts from the marginal effects of interaction between perceived risk to vulnerable and country with 95% CI for the perceived risk to vulnerable model with country as a fixed effect in the period between December 2020 and September 2021.

Country	Total participants	Total surveys	Gender Female (%)	Gender Male (%)	Age group 18-29 (%)	Age group 30-39 (%)	Age group 40-49 (%)	Age group 50-59 (%)	Age group 60-69 (%)	Age group 70-120 (%)	Average survey comp.
Austria	1738	7642	48.4	51.4	18.5	17.3	16.8	19.5	18.5	9.4	4.05
Croatia	1971	7080	51.4	48.6	18.4	16.4	20.0	18.1	21.6	5.4	3.59
Denmark	1730	7057	47.1	52.7	14.1	10.6	13.7	22.4	22.8	16.4	4.07
Estonia	1857	6986	54.0	45.7	13.2	17.0	16.6	18.3	18.6	16.3	3.76
Finland	1620	6444	48.0	51.6	14.8	15.4	15.2	17.0	23.4	14.3	3.97
France	1710	7047	46.4	53.2	15.0	17.4	16.5	15.3	22.4	13.4	4.12
Greece	2245	7130	48.5	51.2	16.1	21.2	26.3	24.4	10.0	2.0	3.18
Hungary	2085	6421	47.4	52.3	12.9	17.1	22.1	16.4	22.2	9.4	3.07
Italy	1617	7054	46.6	53.3	15.0	13.5	21.8	15.7	20.5	13.4	4.36
Lithuania	1918	6429	54.8	45.2	14.3	17.8	16.6	19.1	22.4	9.8	3.35
Poland	1724	7011	55.6	44.4	18.8	19.3	19.2	18.0	18.9	5.8	4.06
Portugal	1840	7031	45.5	54.3	16.0	16.1	21.8	19.2	18.4	8.5	3.82
Slovakia	1733	7049	47.4	52.6	15.3	20.5	20.5	16.5	19.9	7.2	4.07
Slovenia	1998	7510	56.7	43.2	15.8	20.3	21.4	20.9	18.1	3.5	3.76
Spain	1622	7059	49.5	50.3	14.6	16.9	22.4	17.7	18.8	9.4	4.35
Switzerland	1884	6758	48.7	50.9	15.4	18.4	16.3	20.8	17.2	11.9	3.59

Supplementary Table 3: Summary of the sample characteristics of the countries included in the analysis.

Perceived severity Model			
Predictors	Incidence rate ratios	Confidence intervals (CI)	P-value
household size	1.29**	1.28 - 1.30	< 0.001
Gender[Female]-Ref	-	-	-
Gender[Male]	0.90**	0.88 - 0.93	<0.001
Employment status [full time] - Ref	-	-	-
Employment status [part-time (<34 hours)]	0.96	0.90 - 1.02	0.202
Employment status [full time homemaker]	0.79**	0.73 -0.86	<0.001
Employment status [long term sick or disabled]	0.72**	0.63 - 0.82	<0.001
Employment status [Retired]	0.70**	0.64 - 0.74	<0.001
Employment status [Self employed]	0.96	0.90 - 1.03	0.266
Employment status [Student/pupil]	0.88	0.82 - 0.93	<0.001
Employment status [Unemployed and not looking for a job]	0.72**	0.63 - 0.83	<0.001
Employment status [Unemployed and looking for a job]	0.71**	0.67 - 0.75	<0.001
High risk status [No] - Ref			
High risk status [Yes]	0.98	0.94 - 1.02	0.289
History of infection [Infected] - Ref			
History of infection [Not infected]	1.01	0.93 - 1.09	0.852
History of infection [Not tested]	0.92	0.85 - 0.99	0.026
Vaccination status [Not vaccinated] - Ref			
Vaccination status [vaccinated]	1.31**	1.23 - 1.39	<0.001
Perceived severity [high perception] - Ref	-	-	-
Perceived severity [low perception]	1.25**	1.13 - 1.37	<0.001
Perceived severity [neutral perception]	1.10	1.0 - 1.21	0.091
Day of week[weekday] - Ref			
Day of week[weekend]	0.90	0.86 - 0.93	<0.001
Age group [18-29] - Ref	-	-	-
Age group [30-39]	0.95	0.90 - 1.00	0.072
Age group [40-49]	1.02	0.97 - 1.08	0.424
Age group [50-59]	1.04	0.98 - 1.10	0.117
Age group [60-69]	1.06	0.98 - 0.113	0.129
Age group [70-120]	1.01	0.91 - 1.11	0.772
Stringency index [low] -Ref	-	-	-
Stringency index [Moderate]	0.92	0.85 - 0.95	0.001
Stringency index [High]	0.87**	0.80 - 0.95	0.001
Stringency index [Very high]	0.84**	0.77 - 0.92	<0.001
Vaccination status* Perceived severity [low perception]	0.88**	0.80 - 0.96	<0.001
Vaccination status* Perceived severity [neutral perception]	0.94	0.86 - 1.02	0.152
Age group [18-29]* Perceived severity [low perception] -Ref	-	-	-
Age group [30-39]* Perceived severity [low perception]	0.95	0.82 - 1.09	0.462
Age group [40-49]* Perceived severity [low perception]	0.92	0.80 - 1.05	0.213
Age group [50-59]* Perceived severity [low perception]	0.95	0.82 - 1.10	0.496
Age group [60-69]* Perceived severity [low perception]	0.86	0.74 - 1.01	0.065
Age group [70-120]* Perceived severity [low perception]	1.08	0.87 - 1.33	0.491
Age group [18-29]* Perceived severity [neutral perception] -Ref	-	-	-
Age group [30-39]* Perceived severity [neutral perception]	0.92	0.79 - 1.07	0.293
Age group [40-49]* Perceived severity [neutral perception]	0.92	0.80 - 1.06	0.267
Age group [50-59]* Perceived severity [neutral perception]	0.97	0.83 - 1.12	0.652
Age group [60-69]* Perceived severity [neutral perception]	0.88	0.75 - 1.03	0.111
Age group [70-120]* Perceived severity [neutral perception]	0.90	0.73 -1.11	0.313
Stringency index [low]* Perceived severity [low perception] -Ref	-	-	-
Stringency index [Moderate]* Perceived severity [low perception]	0.85	0.75 - 0.96	0.008
Stringency index [High]* Perceived severity [low perception]	0.88	0.78 - 1.00	0.050
Stringency index [Very high]* Perceived severity [low perception]	0.93	0.84 - 1.04	0.217
Stringency index [low]* Perceived severity [neutral perception] -Ref	-	-	-
Stringency index [Moderate]* Perceived severity [neutral perception]	1.00	0.89 - 1.12	0.982
Stringency index [High]* Perceived severity [neutral perception]	1.02	0.91 - 1.14	0.781
Stringency index [Very high]* Perceived severity [neutral perception]	0.98	0.88 - 1.10	0.723

Supplementary Table 4: Results from the hierarchical generalized linear mixed effects model showing the incidence rate ratios, the associated confidence intervals and p-values for the perceived severity model.

Perceived severity Model			
Variable	Chisq	Df	Pr (> chisq)
Household size	1553.604	1	< 0.001
Gender	43.524	1	<0.001
Employment status	260.414	8	< 0.001
High risk status	1.122	1	0.355
Stringency index	15.463	3	0.001
History of infection	28.794	2	0.001
Age group	17.634	5	0.003
Perceived severity	21.369	2	<0.001
Vaccination status	76.989	1	<0.001
Day of week	33.397	1	<0.001
Stringency index : Perceived severity	8.035	6	0.063
Vaccination status : Perceived severity	8.881	2	0.011

Supplementary Table 5: Analysis of Deviance (Type III Wald tests) for the perceived severity model.

Perceived susceptibility Model			
Predictors	Incidence rate ratios	Confidence intervals (CI)	P-value
household size	1.29**	1.28 - 1.31	< 0.001
Gender[Female]-Ref	-	-	-
Gender[Male]	0.91**	0.88 - 0.93	<0.001
Employment status [full time] - Ref	-	-	-
Employment status [part-time (<34 hours)]	0.96	0.88 - 0.93	0.218
Employment status [full time homemaker]	0.80**	0.73 -0.87	<0.001
Employment status [long term sick or disabled]	0.71**	0.63 - 0.81	<0.001
Employment status [Retired]	0.70**	0.66 - 0.75	<0.001
Employment status [Self employed]	0.97	0.90 - 1.04	0.332
Employment status [Student/pupil]	0.89	0.83 - 0.96	0.001
Employment status [Unemployed and not looking for a job]	0.73**	0.64 - 0.84	<0.001
Employment status [Unemployed and looking for a job]	0.71**	0.67 - 0.76	<0.001
High risk status [No] - Ref			
High risk status [Yes]	0.95	0.92 - 0.99	0.006
History of infection [Infected] - Ref			
History of infection [Not infected]	1.01	0.94 - 1.09	0.785
History of infection [Not tested]	0.92	0.86 - 1.00	0.040
Vaccination status [Not vaccinated] - Ref			
Vaccination status [vaccinated]	1.22**	1.17 - 1.27	<0.001
Perceived susceptibility [high perception] - Ref	-	-	-
Perceived susceptibility [low perception]	0.91	0.83 - 0.99	0.022
Perceived susceptibility [neutral perception]	0.96	0.88 - 1.04	0.321
Day of week[weekday] - Ref			
Day of week[weekend]	0.89**	0.86 - 0.93	<0.001
Age group [18-29] - Ref	-	-	-
Age group [30-39]	0.95	0.90 - 1.00	0.045
Age group [40-49]	1.01	0.96 - 1.07	0.587
Age group [50-59]	1.03	0.98 - 1.09	0.252
Age group [60-69]	1.05	0.98 - 1.12	0.178
Age group [70-120]	1.02	0.93 - 1.11	0.745
Stringency index [low] -Ref	-	-	-
Stringency index [Moderate]	0.89	0.81 - 0.98	0.019
Stringency index [High]	0.82**	0.75 - 0.90	<0.001
Stringency index [Very high]	0.80**	0.72 - 0.87	<0.001
Stringency index [low]* Perceived susceptibility [low perception] -Ref	-	-	-
Stringency index [Moderate]* Perceived susceptibility [low perception]	0.91	0.80 - 1.02	0.109
Stringency index [High]* Perceived susceptibility [low perception]	1.03	0.92 - 1.16	0.584
Stringency index [Very high]* Perceived susceptibility [low perception]	0.95	0.85 - 1.06	0.323
Stringency index [low]* Perceived susceptibility [neutral perception] -Ref	-	-	-
Stringency index [Moderate]* Perceived susceptibility [neutral perception]	1.07	0.95 - 1.21	0.251
Stringency index [High]* Perceived susceptibility [neutral perception]	1.06	0.95 - 1.18	0.308
Stringency index [Very high]* Perceived susceptibility [neutral perception]	1.06	0.95 - 1.17	0.315

Supplementary Table 6: Results from the hierarchical generalized linear mixed effects model showing the incidence rate ratios, the associated confidence intervals and p-values for the perceived susceptibility model.

Perceived susceptibility Model			
Variable	Chisq	Df	Pr (> chisq)
Household size	1555.942	1	< 0.001
Gender	41.170	1	<0.001
Employment status	255.091	8	< 0.001
High risk status	7.503	1	0.015
History of infection	26.573	2	<0.001
Stringency index	24.602	3	<0.001
Age group	16.808	5	0.004
Perceived susceptibility	5.381	2	0.067
Vaccination status	96.101	1	<0.001
Day of week	34.591	1	<0.001
Stringency index : Perceived susceptibility	13.242	6	0.039

Supplementary Table 7: Analysis of Deviance (Type III Wald tests) for the perceived susceptibility model.

Perceived risk to vulnerable Model			
Predictors	Incidence rate ratios	Confidence intervals (CI)	P-value
household size	1.30**	1.28 - 1.31	< 0.001
Gender[Female]-Ref	-	-	-
Gender[Male]	0.91**	0.88 - 0.94	<0.001
Employment status [full time] - Ref	-	-	-
Employment status [part-time (<34 hours)]	0.97	0.91 - 1.03	0.267
Employment status [full time homemaker]	0.79**	0.73 -0.86	<0.001
Employment status [long term sick or disabled]	0.72**	0.63 - 0.82	<0.001
Employment status [Retired]	0.70**	0.66 - 0.74	<0.001
Employment status [Self employed]	0.96	0.90 - 1.03	0.300
Employment status [Student/pupil]	0.88	0.82 - 0.95	0.001
Employment status [Unemployed and not looking for a job]	0.73**	0.63 - 0.83	<0.001
Employment status [Unemployed and looking for a job]	0.71**	0.67 - 0.76	<0.001
High risk status [No] - Ref			
High risk status [Yes]	0.95	0.92 - 0.98	0.005
History of infection [Infected] - Ref			
History of infection [Not infected]	1.00	0.93 - 1.08	0.918
History of infection [Not tested]	0.92	0.85 - 0.99	0.028
Vaccination status [Not vaccinated] - Ref			
Vaccination status [vaccinated]	1.21**	1.17 - 1.26	<0.001
Perceived risk to vulnerable [high perception] - Ref	-	-	-
Perceived risk to vulnerable [low perception]	0.95	0.91 - 0.99	0.010
Perceived risk to vulnerable [neutral perception]	0.86	0.83 - 0.90	< 0.001
Day of week[weekday] - Ref			
Day of week[weekend]	0.89**	0.86 - 0.93	<0.001
Age group [18-29] - Ref	-	-	-
Age group [30-39]	0.95	0.90 - 1.00	0.057
Age group [40-49]	1.02	0.96 - 1.07	0.522
Age group [50-59]	1.04	0.98 - 1.10	0.206
Age group [60-69]	1.06	0.99 - 1.13	0.114
Age group [70-120]	1.02	0.93 - 1.12	0.631
Stringency index [low] -Ref	-	-	-
Stringency index [Moderate]	0.88	0.84 - 0.93	< 0.001
Stringency index [High]	0.84**	0.79 - 0.90	<0.001
Stringency index [Very high]	0.80**	0.75 - 0.86	<0.001

Supplementary Table 8: Results from the hierarchical generalized linear mixed effects model showing the incidence rate ratios, the associated confidence intervals and p-values for the perceived risk to vulnerable model.

Perceived risk to vulnerable Model			
Variable	Chisq	Df	Pr (> chisq)
Household size	1572.45	1	< 0.001
Gender	36.763	1	<0.001
Employment status	260.174	8	< 0.001
High risk status	7.879	1	0.005
History of infection	26.322	2	<0.001
Stringency index	48.940	3	<0.001
Age group	17.560	5	< 0.001
Perceived risk to vulnerable	49.083	2	< 0.001
Vaccination status	91.285	1	<0.001
Day of week	34.640	1	<0.001

Supplementary Table 9: Analysis of Deviance (Type III Wald tests) for the perceived risk to vulnerable model.

Perceived severity Model			
Predictors (Contact away from home)	Incidence rate ratios	Confidence intervals (CI)	P-value
household size	1.30**	1.28 - 1.31	< 0.001
Gender[Female]-Ref	-	-	-
Gender[Male]	0.90**	0.88 - 0.93	<0.001
Employment status [full time] - Ref	-	-	-
Employment status [part-time (<34 hours)]	0.97	0.90 - 1.03	0.315
Employment status [full time homemaker]	0.79**	0.73 -0.86	<0.001
Employment status [long term sick or disabled]	0.71**	0.62 - 0.81	<0.001
Employment status [Retired]	0.69**	0.65 - 0.74	<0.001
Employment status [Self employed]	0.96	0.89 - 1.03	0.248
Employment status [Student/pupil]	0.87	0.81 - 0.94	< 0.001
Employment status [Unemployed and not looking for a job]	0.71**	0.62 - 0.81	<0.001
Employment status [Unemployed and looking for a job]	0.70**	0.66 - 0.74	<0.001
High risk status [No] - Ref			
High risk status [Yes]	0.98	0.94 - 1.02	0.354
History of infection [Infected] - Ref			
History of infection [Not infected]	0.99	0.92 - 1.06	0.885
History of infection [Not tested]	0.91	0.84 - 0.98	0.021
Vaccination status [Not vaccinated] - Ref			
Vaccination status [vaccinated]	1.31**	1.23 - 1.39	<0.001
Perceived severity [high perception] - Ref	-	-	-
Perceived severity [low perception]	1.30**	1.16 - 1.47	<0.001
Perceived severity [neutral perception]	1.10	0.97 - 1.26	0.144
Day of week[weekday] - Ref			
Day of week[weekend]	0.90	0.87 - 0.93	<0.001
Age group [18-29] - Ref	-	-	-
Age group [30-39]	1.00	0.91 - 1.10	0.982
Age group [40-49]	1.07	0.97 - 1.17	0.160
Age group [50-59]	1.06	0.97 - 1.16	0.193
Age group [60-69]	1.10	1.00 - 1.22	0.045
Age group [70-120]	1.02	0.91 - 1.14	0.778
Stringency index [low] -Ref	-	-	-
Stringency index [Moderate]	0.92	0.85 - 0.99	0.028
Stringency index [High]	0.87	0.80 - 0.95	0.002
Stringency index [Very high]	0.84**	0.77 - 0.92	<0.001
Vaccination status* Perceived severity [low perception]	0.88	0.80 - 0.96	0.003
Vaccination status* Perceived severity [neutral perception]	0.93	0.85 - 1.02	0.107
Age group [18-29]* Perceived severity [low perception] -Ref	-	-	-
Age group [30-39]* Perceived severity [low perception]	0.93	0.82 - 1.05	0.235
Age group [40-49]* Perceived severity [low perception]	0.91	0.81 - 1.03	0.145
Age group [50-59]* Perceived severity [low perception]	0.97	0.85 - 1.10	0.621
Age group [60-69]* Perceived severity [low perception]	0.92	0.80 - 1.05	0.205
Age group [70-120]* Perceived severity [low perception]	1.14	0.95 - 1.37	0.155
Age group [18-29]* Perceived severity [neutral perception] -Ref	-	-	-
Age group [30-39]* Perceived severity [neutral perception]	0.94	0.83 - 1.08	0.381
Age group [40-49]* Perceived severity [neutral perception]	0.96	0.85 - 1.09	0.557
Age group [50-59]* Perceived severity [neutral perception]	0.99	0.87 - 1.12	0.848
Age group [60-69]* Perceived severity [neutral perception]	0.92	0.80 - 1.05	0.205
Age group [70-120]* Perceived severity [neutral perception]	0.97	0.81 -1.15	0.155
Stringency index [low]* Perceived severity [low perception] -Ref	-	-	-
Stringency index [Moderate]* Perceived severity [low perception]	0.89	0.80 - 0.99	0.032
Stringency index [High]* Perceived severity [low perception]	0.90	0.81 - 1.01	0.083
Stringency index [Very high]* Perceived severity [low perception]	0.88	0.79 - 0.98	0.023
Stringency index [low]* Perceived severity [neutral perception] -Ref	-	-	-
Stringency index [Moderate]* Perceived severity [neutral perception]	1.01	0.90 - 1.13	0.927
Stringency index [High]* Perceived severity [neutral perception]	1.01	0.90 - 1.13	0.929
Stringency index [Very high]* Perceived severity [neutral perception]	0.98	0.87 - 1.09	0.701

Supplementary Table 10: Results from the hierarchical generalized linear mixed effects model showing the incidence rate ratios, the associated confidence intervals and p-values for the perceived severity model for the number of contacts made away from home.

Variable	Estimate	2.5%	97.5%	Standard error	P-value
Intercept	0.347	0.177	0.516	0.086	<0.001
Vaccination status[vaccinated]:Low	0.074	0.002	0.147	0.036	0.042
Country:Austria :Low	-.160	-0.548	0.135	0.151	0.290
Country:Denmark :Low	-0.433	-0.752	-0.154	0.152	0.002
Country:Estonia :Low	0.202	0.038	0.366	0.083	0.015
Country:Finland :Low	-0.224	-0.392	-0.056	0.085	0.008
Country:France :Low	-0.047	-0.313	0.218	0.135	0.726
Country:Greece :Low	0.135	-0.066	0.337	0.103	0.188
Country:Italy :Low	-0.184	-0.664	-0.345	0.081	<0.001
Country:Hungary :Low	-0.504	-0.476	0.108	0.149	0.217
Country:Lithuania :Low	-0.502	-0.659	-0.344	0.080	<0.001
Country:Poland :Low	0.459	0.155	0.763	0.155	0.003
Country:Portugal :Low	0.130	-0.207	0.469	0.172	0.449
Country:Slovakia :Low	-0.372	-0.544	-0.199	0.087	<0.001
Country:Slovenia :Low	0.084	-0.128	-0.297	0.108	0.435
Country:Spain :Low	0.050	-0.292	0.393	0.175	0.774
Country:Switzerland :Low	-0.582	-0.749	-0.415	0.085	<0.001
Gender [male]:Low	-0.063			0.037	0.091
Age group [30-39]:Low	0.152	0.007	0.296	0.073	0.038
Age group [40-49]:Low	0.215	0.075	0.356	0.071	0.002
Age group [50-59]:Low	0.611	0.473	0.748	0.0701	<0.001
Age group [60-69]:Low	1.071	0.936	1.206	0.068	<0.001
Age group [70-120]:Low	1.551	1.396	1.707	0.079	<0.001
Wave[2]:Low	-0.104	-0.164	-0.044	0.0306	<0.001
Wave[3]:Low	-0.137	-0.201	-0.072	0.033	<0.001
Wave[4]:Low	-0.153	-0.2214	-0.085	0.034	<0.001
Wave[5]:Low	-0.244	-0.316	-0.172	0.0366	<0.001
Wave[6]:Low	-0.223	-0.301	-0.144	0.039	<0.001
Wave[7]:Low	-0.323	-0.429	-0.217	0.054	<0.001
Vaccination status[vaccinated]:Neutral	0.152	0.060	0.245	0.047	0.001
Country:Austria :Neutral	0.599	0.292	0.906	0.156	< 0.001
Country:Denmark :Neutral	0.212	-0.117	0.542	0.168	0.206
Country:Estonia :Neutral	0.237	0.036	0.437	0.102	0.020
Country:Finland :Neutral	0.412	0.224	0.600	0.095	< 0.001
Country:France :Neutral	-0.091	-0.392	0.210	0.153	0.553
Country:Greece :Neutral	-0.118	-0.376	0.139	0.131	0.368
Country:Italy :Neutral	0.060	-0.284	0.406	0.176	0.729
Country:Hungary :Neutral	0.098	-0.087	0.284	0.094	0.298
Country:Lithuania :Neutral	-0.388	-0.579	-0.1989	0.097	< 0.001
Country:Poland :Neutral	-0.095	-0.458	0.268	0.185	0.607
Country:Portugal :Neutral	-0.761	-1.265	-0.258	0.257	0.003
Country:Slovakia :Neutral	0.400	0.205	0.595	0.099	< 0.001
Country:Slovenia :Neutral	0.142	-0.111	0.397	0.129	0.270
Country:Spain :Neutral	0.020	-0.359	0.401	0.194	0.914
Country:Switzerland :Neutral	0.619	0.433	0.806	0.095	< 0.001
Gender [male]:Neutral	0.160	0.079	0.242	0.041	< 0.001
Age group [30-39]:Neutral	-0.404	-0.541	-0.268	0.069	< 0.001
Age group [40-49]:Neutral	-0.526	-0.661	-0.391	0.068	< 0.001
Age group [50-59]:Neutral	-0.728	-0.865	-0.592	0.069	< 0.001
Age group [60-69]:Neutral	-0.870	-1.010	-0.731	0.070	< 0.001
Age group [70-120]:Neutral	-0.640	-0.807	-0.473	0.085	< 0.001
Wave[2]:Neutral	-0.036	-0.112	0.039	0.038	0.350
Wave[3]:Neutral	-0.043	-0.124	0.036	0.041	0.287
Wave[4]:Neutral	-0.056	-0.142	0.028	0.043	0.193
Wave[5]:Neutral	-0.051	-0.141	0.038	0.046	0.264
Wave[6]:Neutral	-0.081	-0.179	0.017	0.050	0.106
Wave[7]:Neutral	-0.098	-0.234	0.037	0.069	0.154

Supplementary Table 11: Results from the generalized estimating equation model for the perceived severity for the estimated log odds of estimates and their associated 95% CI of the model variables.
