# Supplementary material for: The influence of COVID-19 risk perception and vaccination status on the number of social contacts across Europe: insights from the CoMix study
Source: BMC Public Health. 2023 Jul 13;23:1350. doi: 10.1186/s12889-023-16252-z (PMC10347859; doi:10.1186/s12889-023-16252-z)
Supplement: Supplementary file 2 — Additional file 2. [file 12889_2023_16252_MOESM2_ESM.pdf]

Additional file 2 for: “The influence of COVID-19 risk perceptions and vaccination status on the number of social contacts across Europe: insights from the CoMix study.”

James Wambua[1*], Neilshan Loedy[1], Christopher I Jarvis[2], Kerry LM Wong[2], Christel Faes[1], Rok Grah[3], Bastian Prasse[3], Frank Sandmann[3], Rene Niehus[3], Helen Johnson[3**], W.John Edmunds[2], Philippe Beutels[4,5], Niel Hens[1,4], Pietro Coletti[1]

1 Data Science Institute, I-BioStat, Hasselt University, Hasselt, Belgium.

2 Centre for Mathematical Modelling of Infectious Diseases, Department of Infectious Disease Epidemiology, London School of Hygiene and Tropical Medicine, Keppel Street, WC1E 7HT London, UK

3 European Centre for Disease Prevention and Control (ECDC), Boulevard 40,169 73 Solna, Sweden

4 Centre for Health Economics Research and Modelling Infectious Diseases, Vaccine & Infectious Disease Institute, University of Antwerp, Antwerp, Belgium.

5 The University of New South Wales, School of Public Health and Community Medicine, Sydney, NSW 2033, Australia

** Current address: Health Emergency Preparedness and Response Authority (HERA), European Commission, 1049 Brussels, Belgium.

* Corresponding author: james.wambua@uhasselt.be

1 Additional file 2: Details of country-level ethical approvals or waivers.

This additional file provides details for the country-level ethical approvals, or waivers, for the protocol and questionnaires used in the countries included in the analysis.

Table S12: Details of the country-level ethical approvals, or waivers, for the countries included in the analysis based on the CoMix study questionnaires and protocol. The table has been adapted from Verelst et al. 2021 [1].

Country	Coordinating organization(s)	Ethical Committee	Decision	Date	Reference
Austria	Austrian National Public Health Institute	Ethikkommission der Stadt Wien	Waived (Drugs Act, Medical Devices Act and Vienna Hospital Act not applicable)	15/06/2020	MA 15- EK/20-149-VK NZ
Denmark	Aarhus University	The Central Denmark Region Committees on Health Research Ethics	Waived (not considered as health research study)	30/06/2020	Request 175 / 2020
France	Centre Hospitalier Universitaire de Poitiers	DCRI Centre Hospitalier Universitaire de Poitiers	Waived (not considered a `recherche impliquant la personne humaine telles que dèfinies par l'article R1121-1 du Code de la Santè Publique').	20/08/2020	not applicable
Poland	National Institute of Public Health - National Institute of Hygiene	Komisja Bioetyczna Narodowego Instytutu Zdrowia Publicznego Panstwowego Zaldadu Higieny, Warszawa	Approved	26/01/2021	Opinia nr 5/2020 z dnia 06.08.2020 r.
Portugal	Instituto Nacional de Saude Doutor Ricardo Jorge (INSA)	Comissão de Ètica para a Saúde do Instituto Nacional de Sa_ude Doutor Ricardo Jorge	Approved	23/06/2020	not applicable
Spain	Institut de Investigació en Ci`encies de la Salut Germans Trias I Pujol	Comit`e dÈtica de la Investigación del Hospital Universitari Germans Trias i Pujol	Approved	04/09/2020	PI-20-242
Italy	ISI Foundation	Comitato di Bioetica d'Ateneo, Universitá degli studi di Torino	Approved	20/10/2020	d65925
Finland	Finnish Institute for Health and Welfare (THL)	Institutional Review Board of the Finnish Institute for Health and Welfare	Waived (Medical Research Act not applicable)	18/12/2020	not applicable
Switzerland	University of Bern	Gesundheits-, Sozial- und Integrationsdirektion Kantonale Ethikkommission für die Forschung	Approved	21/12/2020	2020-02926
Lithuania	Vilnius University	LITHUANIAN BIOETHICS COMMITTEE	Waived (Law on Ethics of Biomedical Research of the Republic of Lithuania not applicable)	20-11-2020	No. 6B-20-298/1
Greece	University of West Attica (UniWA)	Research Ethics Committee (E.H.D.E.)University of West Attica (PADA)	Approved	25/01/2021	4857 / 21-01-2021
Slovenia	National Institute of Public Health	Komisija Republike Slovenije za medicinsko etiko (KME RS)	Approved	29/12/2020	0120-534/2020/14
Croatia	Croatian Institute of Public Health (CIPH)	Etiko povjerenstvo Hrvatskog zavoda za javno zdravstvo (HZJZ)	Approved	16/02/2021	Ur. broj: 381-15-21-2
Estonia	National Institute for Health Development	Research Ethics Committee of the National Institute for Health Development	Approved	02/03/2021	673
Hungary	University of Szeged	Health Science Council Scientific and Research Ethics Committee (ETT TUKEB)	Approved	21/04/2021	IV/3075- 1 /2021/EKU
Slovakia	Comenius University in Bratislava	Eticka komisia Univerzita Komenskeho v Bratislave Jesseniova lekarska fakulta v Martine	Approved	18/05/2020	EK 2/2021

References

1. Verelst F, Hermans L, Vercruysse S, Gimma A, Coletti P, Backer JA, Wong KL, Wambua J, van Zandvoort K, Willem L, Bogaardt L. SOCRATES-CoMix: a platform for timely and open-source contact mixing data during and in between COVID-19 surges and interventions in over 20 European countries. BMC medicine. 2021 Dec;19(1):1-7.
